# Quantitative Assessment of Stereotyped and Challenged Locomotion after Lesion of the Striatum: A 3D Kinematic Study in Rats

**DOI:** 10.1371/journal.pone.0007616

**Published:** 2009-10-27

**Authors:** Olivier Perrot, Davy Laroche, Thierry Pozzo, Christine Marie

**Affiliations:** INSERM U887 Motricité-Plasticité, Université de Bourgogne, Dijon, France; National Institutes of Health, United States of America

## Abstract

**Background:**

Although the striatum is in position to regulate motor function, the role of the structure in locomotor behaviour is poorly understood. Therefore, a detailed analysis of locomotion- and obstacle avoidance-related parameters was performed after unilateral lesion of the striatum in rats.

**Methods and Results:**

Using the three dimensional motion capture technology, kinematics of walking and clearing obstacles, head and body orientation were analyzed before and up to 60 days after the lesion. Recordings were performed in treadmill running rats with or without obstacles attached to the treadmill belt. The lesion, which was induced by the direct injection of the mitochondrial toxin malonate into the left caudoputamen resulted in the complete destruction of the dorsal striatum. During the first three days following the lesion, rats were unable to run on the treadmill. Thereafter, rats showed normal looking locomotion, yet the contralesional limbs exhibited changes in length and timing parameters, and were overflexed. Moreover, the head of lesioned rats was orientated towards the side of the lesion, and their postural vertical shifted towards the contralesional side. During obstructed running, the contralesional limbs when they were leading the crossing manoeuvre stepped on the obstacle rather than to overcome obstacle without touching it, yet more frequently with the forelimb than the hindlimb. Unsuccessful crossings appeared to be due to a paw placement farther away from the front of the obstacles, and not to an inappropriate limb elevation. Importantly, deficit in locomotor behaviour did not regress over the time.

**Conclusion:**

Our results argue that the striatum of one hemisphere controls kinematics of contralateral limbs during stereotyped locomotion and plays a prominent role in the selection of the right motor program so that these limbs successfully cross over obstacle.

## Introduction

The study of quadrupeds has furnished most of our understanding of mammalian locomotion [1], [2]. Thus, locomotion is controlled by the interaction of three components: (1) central pattern generators (CPGs), networks of spinal interneurons which provide the basic locomotor pattern, (2) proprioceptive and exteroceptive feedbacks, and (3) descending supraspinal control from the brain cortex including the corticospinal pathway and from the brain stem including the rubro-, vestibulo- and tecto-spinal pathways. The cortico- and rubro-spinal tracts are responsive for fine control and voluntary modification of locomotion and the other tracts serve to activate CPGs, which are silent at rest, and to adjust the posture. However, how these components are implemented and how they interplay to generate/regenerate locomotion in normal/pathological conditions is not well understood.

The striatum is the main input layer of the basal ganglia. It is organized in three zones; the sensorimotor, the associative and the limbic zones, which receives afferents from the sensori-motor, associative, and the limbic cortical areas, respectively [3]. The commonest consequence of lesion of the striatum is dystonia and the syndrome of abulia (apathy with loss of initiative and of spontaneous thought and emotional response) in human depending on the site of the lesion within the striatum [4]. However, electrophysiogical studies in primates and imaging studies in humans are in keeping with the idea that the striatum supports hand/fingers movement selection, preparation and execution [5]–[9]. In contrast, the role of the striatum in the regulation of locomotion and the voluntary adaptation of locomotion to environment, which requires a precise and fine supraspinal control of the basic locomotor pattern, is not well understood. Activation of the striatum during treadmill locomotion in rats [10] and during the imagination of locomotor tasks in human [11] has been reported. However, most of what is known of the role of the striatum in the control of locomotion has been deduced from the disturbances of gait accompanying Parkinson's disease (PD) including slow gait speed, little steps, narrowing of base support and lack of swing arm, but normal performance in obstacle avoidance tasks [12]. Assuming that PD is caused by striatal dopamine depletion consecutive to degeneration of dopaminergic neurons originating from the substantia nigra (SN) pars compacta, locomotor deficit in PD only reveals how important is the striatal dopaminergic input in the control of the basic locomotor pattern. Additionally, the functional deficit observed in PD patients is the net result of two opposite phenomena, i.e. the severity of the striatal dopamine depletion and the intensity of the different compensatory mechanisms, which are sequentially activated in parallel with the progressive striatal dopamine depletion [13]. A detailed and quantitative analysis of stereotyped and challenged locomotion after acute lesion of the striatum in rat may help to increase our understanding on the role of the striatum in locomotor behaviour. It could also help to interpret the locomotor deficit and recovery observed in stroke patients in which the striatum alone or in combination with cortical areas is a common site of acute neuronal death.

The aim of the present study was to better understand the role of the striatum in the two components of the locomotion; the basic locomotor pattern which is provided by CPGs, and the possibility to deal with environmental constraints by the voluntary modification of the basic locomotor pattern, which requires supraspinal control. For this purpose, locomotion was studied before and up to two months after unilateral lesion of the striatum during treadmill running with or without obstacles attached to the treadmill belt in the rat. The brain lesion was induced by the direct injection of the mitochondrial toxin malonate into the dorsal striatum (caudoputamen) which includes the sensorimotor and the associative zones of the striatum in rat. Locomotion was quantitatively and objectively assessed from the 3-D motion capture technology. Recordings were also performed in sham rats in order to assess the impact of the surgical procedure on kinematics.

## Results

In a first experiment (10 rats with body weight about 450 g), 8 rats were selected at the end of the selection period, 1 rat died during anaesthesia, and the remaining rats were treated with malonate. In lesioned rats, pre-lesion kinematic recordings were performed 19, 12, 5 and 1 days before malonate administration. During this period, the body weight (g) increased from 467±16 to 478±18. Pre-lesion kinematic parameters were not different. Therefore, the values were pooled and compared to values collected 4, 7, 14, 21, 28, 35, 42, 49, or 60 days after malonate administration (n = 7 at all time points except at day 4 lesion where n = 6). During the post-lesion period, the body weight (g) increased from 431±14 to 499±15. The weight loss was not due to some difficulties to reach food.

In a second experiment (6 rats with a body weight of 370 g), 4 rats were selected at the end of the selection period and treated with saline instead of malonate (sham rats). Among these rats, one rat was excluded from the kinematic analysis because of frequent removals of the forelimb distal markers with teeth. Kinematic recordings were performed 3, 2 and 1 days before saline administration. As values were not different, they were pooled and compared to values collected 1, 2 and 4 days after saline administration. In sham rats, the body weight remained close to 370 g.

### 1) Histological study

**Figure 1:**
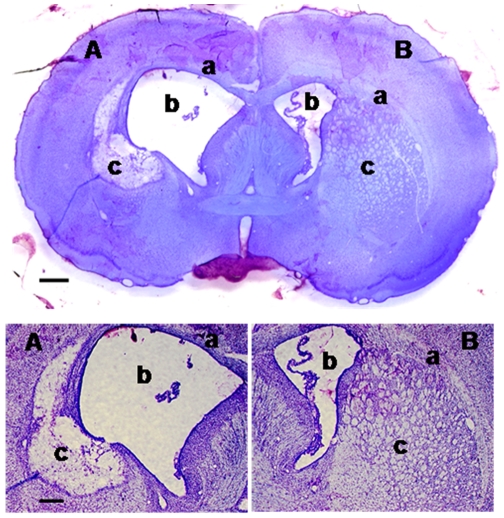
Representative photographs of a brain slice passing through the lesioned striatum. Note the preservation of the corpus callosum (a), the dilation of the lateral ventricle (b) and the cavities within the striatum (c) of the lesioned side (A) as compared to the unlesioned site (B) Staining: cresyl violet; scale bars for the top and the bottom photographs are 350 and 500 µm, respectively.

After malonate administration, all rats exhibited a complete lesion of the dorsal striatum which was associated with a severe atrophy of the lesioned hemisphere. The mean lesion volume was 18.9±5.4 mm^3^ and tissue loss in the lesioned hemisphere reached 16.6±4.0% (relative to the unlesioned hemisphere). These data are in accordance with previous data of our laboratory [14]. Fig. 1 shows a representative slice of brain collected at the striatum level. Note the dilation of the ventricle on the lesion side as well as the multiple cavities within the lesioned striatum.

### 2) Overview

Rats were all unable to run on the treadmill during the first three days following malonate administration. At day 4 post-lesion, running was possible in 6 rats, the remaining rat being capable of running from day 7 post-lesion. The running incapacity was apparently due to the inability of rats to adapt limb motion to the treadmill belt movement. Once capable of running, lesioned rats badly performed the obstacle clearance task with their contralesional limbs when these limbs were leading the obstacle manoeuvre. In contrast, sham rats were capable of treadmill running as soon as the first day following saline treatment, and saline did not impair obstacle crossing. Accordingly, changes in locomotor behaviour observed in malonate-treated rats were not due to the surgical procedure. Moreover, deficit after malonate cannot involve changes in body weight. First, kinematic changes were restricted to limbs contralateral to the lesion. If the impairments had been due to changes in body weight, kinematics would have been impaired bilaterally. In addition, changes in kinematics did not parallel with changes in body weight in lesioned rats.

### 3) Effect of the striatal lesion on stereotyped locomotion

Whereas the sham procedure affected none of the measured parameters (data not shown), malonate administration impaired many locomotion-related parameters either transiently or persistently as described in the following paragraphs.

#### a) Timing and length parameters

**Figure 2:**
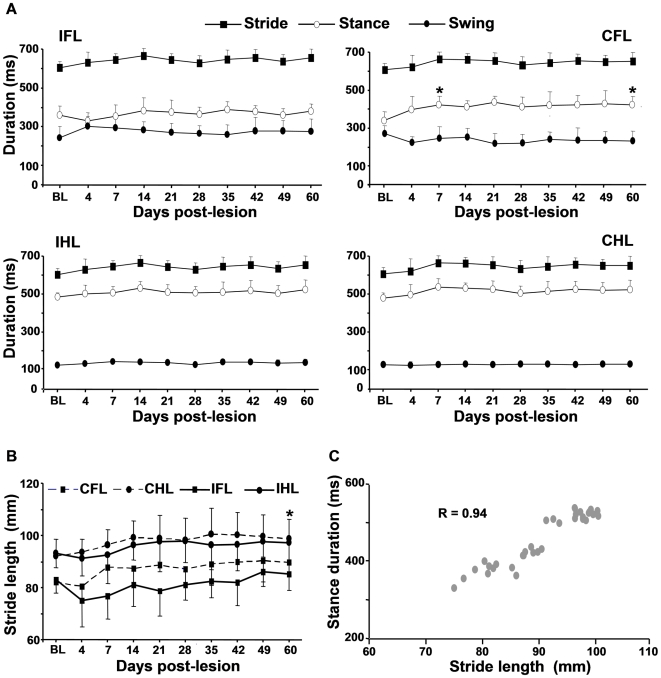
Effect of the lesion on timing and length parameters. A) duration of the stride, the stance and swing phases, B) stride length, C) relationship between the stride length and the stance phase duration after lesion. The parameters were measured before lesion (BL) and up to 60 days after lesion from the ipsilesional and contralesional forelimbs (IFL and CFL) and hindlimbs (IHL and CHL). The stride length was plotted against the corresponding stance phase duration. Values are means±SD, * different from BL values (P<0.025).

After lesion, timing parameters were affected only for the contralesional forelimb (CFL) (Fig. 2A). That limb exhibited an early and persistent increase in the stance phase duration. However, the stride duration remained close to pre-lesion values. The lesion also led to a persistent decrease in temporal symmetry ratio (TSR) between the forelimbs (1.09±0.81 and 0.93±0.37 at day 7 and 60 post-lesion vs 1.66±0.89 before lesion, P<0.025), whereas TSR between the hindlimbs was not modified (1.01±0.31 and 1.02±0.22 at day 7 and 60 post-lesion vs 1.09±0.10 before lesion, NS). Length parameters were also significantly affected by the lesion (Fig. 2B). After lesion, stride length of the contralesional hindlimb (CHL) progressively increased over the time (P<0.025 at day 60 post-lesion). Nevertheless, the stride length remained in proportion with the stance phase duration after lesion (Fig. 2C). Finally, the homologous, homolateral and diagonal coupling between limbs before lesion were ∼50%, 30% and 80%, respectively, and were not affected by the lesion, regardless of the post-lesion time (data not shown).

#### b) Joint angle values

The mean distance between the hip and the knee markers as well as between the knee and the ankle markers was not significantly different between the two hindlimbs and not affected by the lesion. In addition, the distance was not different among rats whatever the time point of the measurement. Therefore, comparisons between pre- and post-lesion values of knee angle values did well inform on the impact of the lesion on joint kinematics.

**Figure 3:**
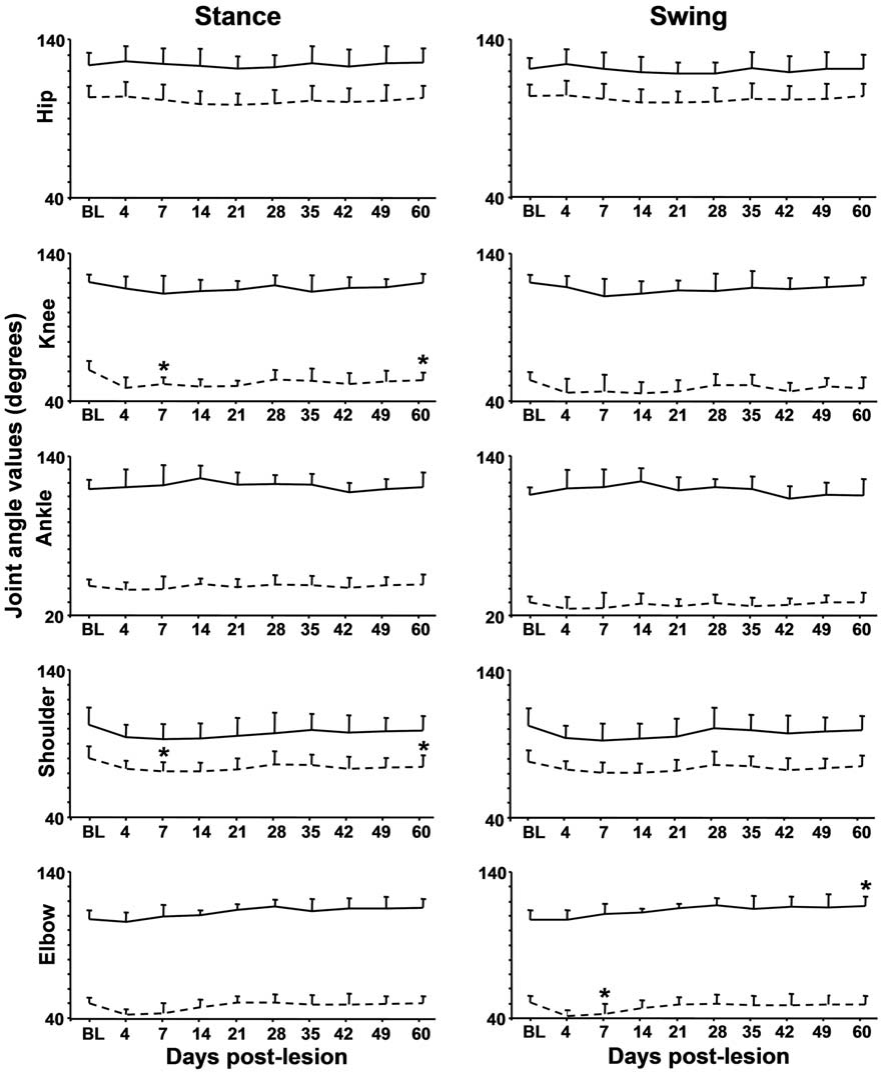
Effect of the lesion on joint angle values of the contralesional limbs. The solid and dashed lines correspond to the maximal and minimal values, respectively. The parameters were measured during the stance and swing phases before lesion (BL) and up to 60 days after lesion. Values are means±SD, * different from BL values (P<0.025).

**Figure 4:**
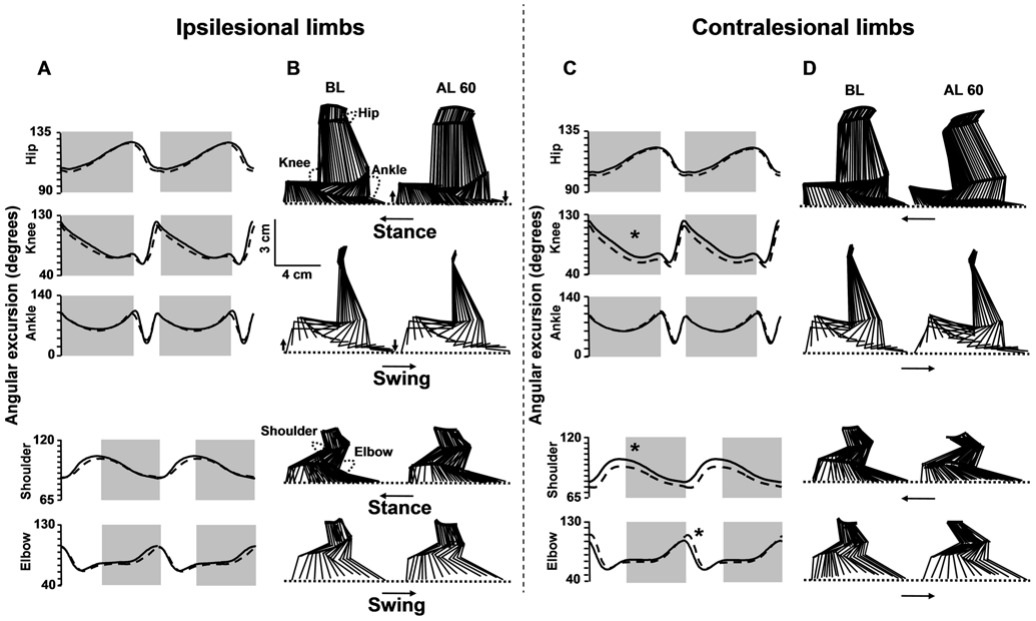
Effect of the lesion on mean values of the joint angle positions. A and C) angular excursion, the solid and dashed lines correspond to values measured before and at day 60 after lesion, respectively. The phases of the locomotor cycle were normalized (the stance phase in grey), * difference between pre- and post-lesion values (P<0.025), B and D) corresponding stick figures of one complete step cycle (stance ad swing). Horizontal arrows indicate the direction of the movement, downward arrows foot contact and upward arrows foot lift.

The impact of the lesion on joint angle values is shown in Fig. 3 and 4. Unlike ipsilesional limbs whose joint angle values were not affected by the lesion, the contralesional limbs exhibited significant changes in joint angle values (Fig. 3). After lesion, the knee and shoulder angles were decreased early and persistently during the stance phase, i.e. the joints were over-flexed. In contrast, a transient over-flexion followed by a delayed over-extension of the elbow was observed during the swing phase. The mean pre- and post-lesion (at day 60) angular excursion (over 2 consecutive cycles) as well as sticks diagrams of a representative rat is shown in Fig. 4. Clearly, the pre-lesion and post-lesion angular traces are superimposed for the ipsilesional but not the contralesional limbs. For these limbs, the post-lesion trace is below the pre-lesion trace for the knee and the shoulder and above the pre-lesion trace for the elbow.

#### c) Paw placement in the frontal plane

**Figure 5:**
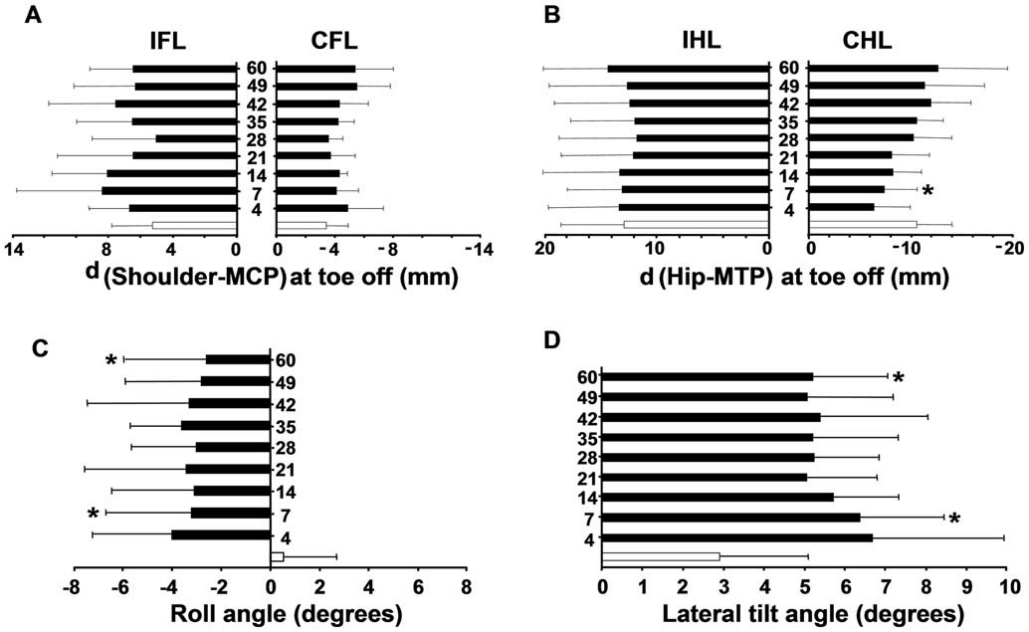
Effect of the lesion on paw placement in the frontal plane and head and body orientation. A) paw placement of forelimbs, B) paw placement of the hindlimbs, C) horizontal head-on-trunk position, D) the lateral shift of the body. Positive values indicate deviation towards the contralesional side and negative values towards the ipsilesional side. IFL, IHL  =  ipsilesional forelimb, hindlimb; CFL, CHL  =  contralesional forelimb, hindlimb. Empty bars represent pre-lesion values and black bars post-lesion values (from day 4 to 60 post-lesion). Values are means±SD, * different from pre-lesion values (P<0.025).

The results are summarized in Fig. 5A and 5B. The lesion resulted in a more internal placement of the contralesional hindlimb. Thus, the distance (mm) between the hip and MTP markers in the frontal plane at toe off was −10.5±3 before lesion and decreased to −6.4±3.5 and −7.4±3.2 at days 4 and 7 post-lesion, respectively. The distance progressively recovered pre-lesion values over the time. The paw placement of other limbs was not affected by the lesion. These data are consistent with a reversible decrease in the hindlimb base of support in the lesioned rats.

#### d) Head and body orientation

The results are summarized in Fig. 5C and 5D. Before lesion, the mean horizontal head-on-trunk position was close to the mid-sagittal body axis as evidenced by the value of the roll angle (0.5±2.2°). A substantial and long-lasting deviation toward the ipsilesional side was observed in lesioned rats. Thus, as shown in Fig. 5C, the roll angle was −3.2.0±3.5° and −2.6±3.0° at days 7 and 60 post-lesion, respectively. In addition, the lesion produced a persistent shift of the body towards the side opposite to the lesion (Fig. 5D) as evidenced by the increased lateral shift angle from day 7 (6.4±2.1°) to day 60 post-lesion (5.2±8°) as compared to pre-lesion values (2.9±2.1°). Such a shift of the body is consistent with the increased flexion of the contralesional limbs (Fig. 3).

### 4) Effect of the striatal lesion on obstacle avoidance

**Figure 6:**
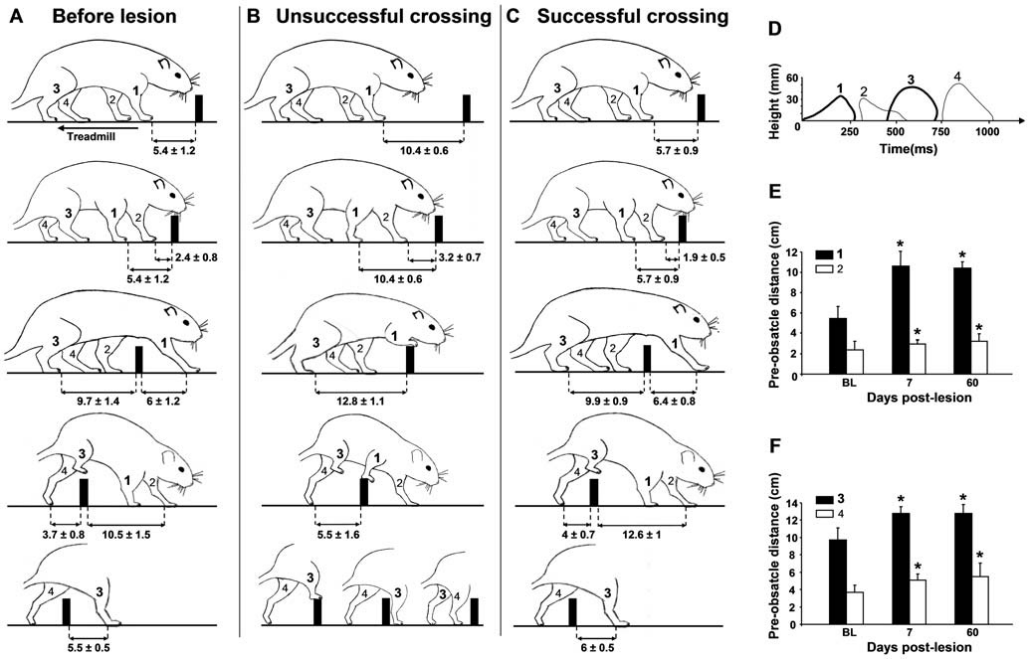
Obstacle avoidance-related parameters. A) before lesion, rats never touch the obstacle and used a strategy in which the first (leading) hindlimb (limb 3) to step over obstacle is always ipsilateral to the leading forelimb (limb 1), B) when the contralesional leading forelimb (limb 1) is placed farther away from the front of the obstacle, it steps on the obstacle (unsuccessfull crossing). The ipsilesional trailed forelimb (limb 2) crosses over obstacle normaly whereas the contralesional leading hindlimb (limb 3) either steps on the obstacle or, crosses over obstacle normally with or without an extrastep before crossing (bottom panel), C) when pre-obstacle distance of the contralesional leading forelimb is not different from pre-lesion values, this limb performs obstacle crossing normally (successful crossing). In this situation, the trailed forelimb (limb 2) is placed further behind the obstacle. D) Pre-lesion limb trajectory for a representative rat, E and F) pre-obstacle distances of the forelimbs (E) and the hindlimbs (F) in situation in which the contralesional forelimb badly performs obstacle crossing. * different from BL (before lesion) values (P<0.025). Values of pre- and post-obstacle distances (cm) are mean±SD, in B and C, values correspond to those measured at day 60 post-lesion.

Before lesion, no preference was shown for leading either with the right or with the left forelimb (data not shown) and rats crossed over the obstacle without touching it. When rats stepped over obstacles, they used a strategy in which the first hindlimb to step over the obstacle was always ipsilateral to the leading forelimb as illustrated in Fig. 6A. In the example, the right forelimb (limb 1) was the first limb to step over the obstacle (leading forelimb), followed by the left forelimb (trailed forelimb  =  limb 2). Then, the rat stepped over the obstacle with the right hindlimb (leading hindlimb  =  limb 3) and finally with the left hindlimb (trailed hindlimb  =  limb 4). The pre-obstacle distances (cm) were 5.4±1.2 and 2.4±0.8 for the leading and trailed forelimbs, respectively. The corresponding values for the hindlimbs were 9.7±1.4 and 3.7±0.8. The maximal height (mm) of the more distal marker during the crossing swing was 36.9±1.4 for limb 1, 30.6±1.7 for limb 2, 45.7±2.8 for limb 3 and 50.4±2.7 for limb 4. For both forelimbs, maximal elevation was reached when the tip of limbs was just above the obstacle. On the contrary, maximal elevation of limbs 3 and 4 was reached before and after the tip of the paw had crossed over the obstacle, respectively. Time to avoid obstacle was ∼250 ms for all limbs. Fig. 6D illustrates limbs trajectory before lesion in a representative rat.

After lesion, no preference for leading was observed either with the contralesional or ipsilesional forelimb (data not shown). However, in the situation in which the contralesional forelimb was leading the crossing manoeuvre, obstacle avoidance was impaired. In contrast, all the limbs crossed over obstacle normally when the ispilesional forelimb was the first to encounter the obstacle. The situation in which the contralesional forelimb was the leading limb (limb 1) is illustrated in Fig. 6B and 6C. In this situation, limb 1 either stepped on the obstacle and remained for varying durations on the obstacle (unsuccessful crossings, Fig 6B) or crossed over the obstacle normally (successful crossing, Fig 6C). When limb 1 badly performed obstacle crossing, limb 2 (forelimb ipsilateral to the lesion) and limb 4 (hindlimb ipsilateral to the lesion) successfully crossed over the obstacle. On the contrary, limb 3 (the leading contralesional hindlimb) either stepped on the obstacle, successfully crossed over the obstacle, or took an extra step before passing over the obstacle without touching it (see bottom panel of Fig. 6B). In steps in which the leading contralesional forelimb normally performed obstacle crossing, the other limbs also crossed over obstacle normally (Fig.6C). Deficit in obstacle avoidance did not regress over the time. Indeed, the percentage of unsuccessful crossings with the leading contralesional forelimb was 63.6±12.2% at day 7 post-lesion and 67.3±13.2% at day 60 post-lesion. The corresponding values for the leading contralesional hindlimb were 28.8±11.1% and 24.7±9.5%.

Unsuccessful crossings with the contralesional forelimb were associated with a placement of the limb farther away from the front of the obstacle and not with an inappropriate limb elevation. Indeed, maximal paw elevation of the leading contralesional forelimb was not different from pre-lesion values (not shown). In contrast, pre-obstacle distance of this limb was 10.4±0.6 cm when it stepped on the obstacle (limb 1 in Fig 6B, above panel) whereas distance (5.7±0.9 cm) was not different from pre-lesion value when the limb overcame obstacle normally (limb 1 in Fig. 6C, above panel). The bad placement of limb 1 was accompanied with a bad placement of other limbs which were also placed farther away from the front of the obstacle. However, the increase in pre-obstacle distance was more important for limb 1 (∼ +200% vs ∼ +30% for other limbs) as shown in Fig. 6E and 6F. Finally, in steps in which the contralesional forelimb was leading the crossing manoeuvre with success (Fig. 6C) as in steps in which the ipsilesional forelimb was leading (not shown), the trailed forelimb was placed farther behind the obstacle as compared to limb position before lesion, at least within the acute post-lesion period. Thus, post-obstacle distance of limb 2 when it was the ipsilesional forelimb was 12.5±1 cm at day 7 (P<0.025) and 12.6±1 cm at day 60 (NS, P = 0.027) vs 10.5±1.5 cm before lesion. On the contrary, maximal elevation of limbs 3 and 4 was reached before and after the tip of the paw had crossed over the obstacle, respectively.

## Discussion

The 3-D motion capture technology primarily dedicated to human is a little-used method in rodents. Available studies focussed on kinematics in normal conditions, after spinal lesion or hindlimb [15]–[18]. Using this high-performing technology, our study reveals persistent changes in the basic locomotor pattern and obstacle avoidance performance after lesion of the striatum in rat.

Within the first week following malonate administration, lesioned rats did initiate treadmill locomotion but were unable to adapt limbs motion with the speed of the treadmill belt, thus resulting in treadmill running incapacity. This suggests that the striatal lesion has compromised the interaction of the three components involved in the neural control of locomotion including CPGs, sensory feedback, and descending supraspinal control. Despite the lack of direct link between CPGs and the striatum, CPGs activity may be indirectly dependent on striatal output. The striatum contains GABAergic neurons that inhibit the SN pars reticulata [19], a brain stem area recently demonstrated to exert tonic inhibition of the mesencephalic locomotor region (MLR) [20], which contains the reticulospinal neurons projecting on CPGs. Therefore, the striatal lesion may produce an abnormal MLR inhibition, thus resulting in a delayed production of locomotion [21] as well as troubles of the rhythmic alternations of limbs [20]. Besides, regarding the sensory processing ability of striatal neurons [22], changes in the sensory control of locomotion may contribute to the observed deficit. Consistent with this mechanism, the motor responses to tactile and proprioceptive stimuli on the contralateral limbs are transiently lost after a striatal lesion [14], [23].

Whatever the mechanisms involved in the treadmill running incapacity after a striatal lesion, all rats regained their ability to regularly run on the treadmill from day 7 post-lesion, suggesting that the intact neuronal circuitry can rapidly compensate for the lesioned striatum when the striatum is engaged in the production of the basic locomotor pattern. However, the neuroplasticity of locomotor control mechanisms did not allow a full recovery of the initial locomotor pattern as evidenced by the persistent increase in the stance phase duration and in the stride length of the contralesional forelimb and hindlimb, respectively. These data argue that the integrity of the striatum is required for the structure and the timing of the basic locomotor pattern as suggested by a recent study that specifically examined the relationship between lesion location and gait asymmetry in ambulatory chronic stroke patients [24]. The authors report that lesion to putamen is evident 60% to 80% more frequently in the asymmetrical patients compared to the symmetrical patients. Further studies are needed to elucidate how the striatum contributes to the basic locomotor pattern knowing that hypermetry of the contralesional hindlimb is also observed after unilateral pyramidal tract section [25] but not after lesion of the somatosensory cortex [26] in the rat.

During stereotyped locomotion, lesioned rats showed abnormal posture as evidenced by the persistent lateral tilting of their body towards the side opposite to the lesion as well as the overflexion of their contralesional limbs. These data are in agreement with the emergent theory that the output nuclei of the basal ganglia (the SN pars reticulata, the globus pallidus, the ventral pallidum) keep the brainstem areas that control posture under tonic inhibition [27]–[29]. However, pathological asymmetry of postural muscle tone regulation is not necessarily the cause of the shift of the body. The shift may be alternatively an attempt to align the body with a vertical reference which should be erroneously perceived to be tilted from true earth vertical in lesioned rats. Evidence that the striatum filters information that originates within the parietal cortex, a structure that has a critical role in the perception of the verticality [30] supports this hypothesis. Interestingly, the shift of the body is towards the contralesional side in stroke patients with a striatal lesion [31], but towards the ipsilesional side in hemiparkinsonian rats [32]. Accordingly, lesions of the striatum and striatal dopamine depletion both produce abnormal posture, yet through different mechanisms.

Obstacle avoidance tasks provide an adequate paradigm to explore the possibility to deal with environmental constraints by the voluntary modification of the basic locomotor pattern. To date, information on obstacle avoidance in human and animals (cats only) with a central lesion are scarce. In addition, available studies focussed on the role of the cerebral cortex and the cerebellum. It was demonstrated that the cerebellum and the motor cortex both contribute to adequate paw placement and limb trajectory [33], [34] and that the posterior parietal cortex is rather involved in planning gait modification [35]. The new finding of the present study is that the integrity of the striatum is required to successful obstacle avoidance (as a second subtask added to locomotion), and that intact neuronal circuitry cannot spontaneously compensate for the lesioned striatum when the structure is engaged in challenged locomotion. Our results show that limbs contralateral to the striatal lesion badly perform obstacle crossing from day 4 to 60 post-lesion in the situation in which the contralesional limb is the first to encounter obstacle. The limbs step on the obstacle and remain for varying durations on the obstacle rather than to overcome obstacle without touching it. An asymmetrical deficit in limbs force production appears to be not involved in deficit because contralesional limbs normally crossed over obstacle when they were the second to encounter the obstacle. Alternatively, impaired performance may be related to persistent hemispatial neglect. Actually, the head of lesioned rats was orientated towards the side of the lesion (see also [36] similarly to that observed in hemiparkinsonian animals [37]–[39] and stroke patients (“Prévost's” sign). This abnormal head orientation leads to the neglect of information on the contralesional side [40], [41]. Because visual input is critical to successful obstacle avoidance with the leading limbs [42], the hemispatial neglect of the right side may therefore explain why only the right limbs stepped on the obstacle after lesion of the left striatum. However, the hindlimb that is moved in the absence of direct visual input also badly performs obstacle crossing, suggesting that mechanisms other than hemispatial neglect also contribute to the impaired performance. Of note, impaired performance in obstacle avoidance is observed even in stroke patients without hemispatial neglect [43]. With an effort to identify the causes of unsuccessful crossings, we have measured the position of the leading forelimb with respect to the obstacle as well as its trajectory as measured by the maximal elevation of limbs during the crossing swing. The results clearly show that unsuccessful crossing is associated with increased pre-obstacle distance and not with inappropriate limb trajectory.

These data suggest an important role of the striatum in the planning rather than execution of the voluntary modification of locomotion. The striatum is thought to select which motor programs should be called into action through multiple cortico-striato-pallido-thalamo-cortical loops. However, the hypothesis that striatal lesion-associated impaired obstacle avoidance solely reflects disconnection between the striatum and the cortical areas is unlikely. Indeed, in cats with lesions of the motor or the parietal cortex, the contralesional limbs badly performed obstacle crossing when they are the leading or the trailed limbs [44]–[46], and hemiplegic stroke patients with unilateral cortical lesion exhibit impaired ability to avoid obstacle regardless of whether the avoidance manoeuvre is led by the affected or unaffected leg [47]. Moreover, as patients with PD perform as well as aged matched controls [12] in obstacle avoidance tasks, the disconnection between the striatum and the SN pars compacta cannot either be involved in unsuccessful obstacle crossing observed after lesion of the striatum.

Malonate is considered as a selective neurotoxin. Nevertheless, since the corticospinal fibres course through the striatum in rats, it is relevant to ask whether some aspects of the observed deficits are attributable to impaired structural or functional integrity of these fibres. Against the existence of structural damage is the normal appearance of the fibres after lesion induced by malonate or the other neurotoxin quinolinic acid [48], [49]. However, this does not mean that functionality of the fibres is not impaired. Functionality of the corticospinal tract has never been investigated after malonate lesions but is spared after quinolinic lesions [50]. Regarding the similarities between malonate and quinolinic lesions with respect to histological characterization [51], it is tempting to speculate that functionality of the corticospinal pathway is normal after malonate lesions, and that deficit after malonate lesions is not due to changes in corticospinal outflow. In accordance with this hypothesis, walking performance is not associated with the extent of lesion overlap with the corticospinal tract in stroke patients [52], and differences exist between deficit induced by lesion of the corticospinal tract and that induced by lesion of the striatum. Stereotyped locomotion is possible as soon as the first day following lesion to the corticospinal tract and most impairments in kinematics and ground reaction forces recover rapidly within the first week after operation [25], [53]. In contrast, treadmill running is impossible during the first three days after malonate, and locomotor behaviour is impaired persistently after malonate. In addition, the contralesional forelimb which badly performs obstacle crossing after striatal lesion (our results) was reported to cross over obstacle normally after pyramidal lesions [45]. Nevertheless, further studies are needed to prove that deficit after malonate is not due in part to damage of the corticospinal fibres.

In conclusion, our results argue that the striatum of one hemisphere controls kinematics of contralateral limbs during stereotyped locomotion and plays a prominent role in the selection of the right motor program so that these limbs successfully cross over obstacle. They also suggest that the intact neuronal circuitry cannot spontaneously compensate for the lesioned striatum, at least when the (dorsal) striatum is fully lesioned. Techniques and data described here are likely to be useful for a better comprehension of the neural pathways involved in the regulation of stereotyped and challenged locomotion, and for the guidance of new therapeutic interventions in pathologies associated with impaired gait.

## Materials and Methods

### Animals

Experiments were carried out on Wistar adult male rats (Depré, Saint-Doulchard, France) with age of 13 weeks. All procedures were approved by the ethical committee of the Université de Bourgogne and were conducted according to guidelines of the French department of agriculture (licence n° 21CAE101). Animals kept in ventilated, humidity and temperature-controlled rooms with a 12/12-h light/dark cycle received food and water *ad libitum*. To reduce the animal's stress level, the same operator performed all steps of the experiments.

### Selection of animals

Rats were selected according to their capacity of running regularly on a horizontal treadmill (Bioseb, Vitrolles, France) with the speed of the treadmill belt fixed at 25 cm/s. A 3 min-long running session (first without obstacles and then with obstacles attached to the belt) was given twice a day for seven days. On the first day, mild intensities of foot shocks were used as negative reinforcement to improve performance. Rats that failed to run in a regular way on the treadmill (contact of the forelimbs with the front wall of the treadmill, frequent immobility or gallop) at the end of the selection period were excluded. It is noteworthy that obstacle clearance was not a difficulty for any of the rats.

### Induction of the lesion

A lesion confined to the caudoputamen was induced by the direct injection of the mitochondrial toxin malonate (disodium salt, Sigma, Saint Quentin Fallavier, France) into the left striatum. Briefly, rats were anaesthetized with chloral hydrate (400 mg/kg, i.p.) and positioned in a stereotaxic frame. Injection of malonate (pH 7.4) was performed into the left striatum via a cannula inserted at the following coordinates relative to bregma: AP: 0.5 mm, Lat: 3.5 mm, V: 6 mm from the skull (Paxinos & Watsons' atlas). Injection of malonate (3 µmol) was carried out over 3 min at a rate of 1 µL/min. According to this dosage, the lesion measured at day 1 after malonate poisoning affects the whole caudoputamen [14], [54]. It can be noticed here that the malonate lesion is a pannecrotic lesion and has revealed striking similarities to the lesion induced by ischemic stroke with respect to histological characterization [51].

### Kinematics recordings

The 3D kinematics data were collected using the VICON MX-13 optical motion capture system (Vicon, Oxford, Great Britain) consisting of 6 high-speed digital cameras placed at approximately 0.7 m from the treadmill. Three cameras were placed facing the rat's left side and three other cameras facing the rat's right side, perpendicular to the direction of the movement, thus allowing the simultaneous recording of the two hemi-bodies. Data were collected at a sampling rate of 200 Hz. The image dimension was 1280×1024 pixels. The magnification of the cameras was calibrated to cover the 45 cm length of the treadmill apparatus.

**Figure 7:**
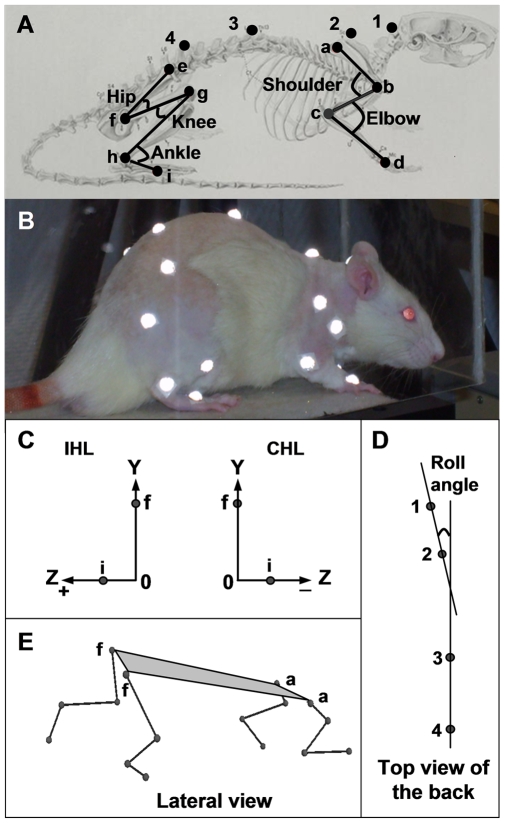
Position of the reflective markers and kinematics parameters in rats. A and B) five markers were placed on each hindlimb, four markers on each forelimb and four markers (1 to 4) on the back, C) the paw placement of the ipsilesional and contralesional hindlimb (IHL, CHL) in the frontal plane was assessed from the position at toe off of the MCP marker (marker i) on the Z-axis (mediolateral) with respect to the Y-axis (vertical) that passed through the hip marker (marker f), D) the horizontal head-on-trunk position was assessed from the roll angle, i.e. the angle between the straight line passing through the dorsal markers 1 and 2 and that passing through the dorsal markers 3 and 4, E) the lateral tilt of the body was assessed from the angle between the plane (in grey) passing through the two hip markers (f) and the two shoulder markers (a) and the horizontal plane of the laboratory (not indicated).

After anaesthesia (chloral hydrate, 400 mg/kg, i.p.) the limbs and the back were shaved and tattooed in order to locate the bony processes as previously described in details [15]. The area around the tattoo marks was regularly shaved and re-touched with permanent ink as soon as tattoo fading was observed. For this step, anaesthesia of animals that were now confident with the experimenter was not required. Twenty two infrared-reflective hemispherical markers (BTS Bioengineering, Cod FMK0005, Milano, Italy) with a diameter of 6 mm were placed over the following anatomical landmarks (see Fig.7A, B): the scapula (marker a), the upper (shoulder marker b) and lower (elbow marker c) humerus epiphysis, the metacarpophalangeal (MTC) joint (marker d), the iliac crest (marker e), the great trochanter (hip marker f), the knee (marker g), the internal malleolus (ankle marker h) and the fifth metatarsophalangeal (MTP) joint (marker i). Four markers (markers 1, 2, 3 and 4) were also placed on the back from the neck to the tail at regular distances. Finally, two markers were placed on the base of each of the two obstacles. Markers were fixed on rat and obstacles with a double face adhesive tape.

The kinematic data were collected with the speed of the treadmill belt fixed at 25 cm/s, a speed that is within the range of speed of rat's overground locomotion [55]. Stereotyped locomotion was first assessed in a 1-min long session (3×20 sec). Then, two obstacles (3 cm high, 1.2 cm wide) separated by 45 cm were attached to the treadmill belt and data were again recorded in a 3-min long session (3×1 min). Soft tissue movement around the knee (skin slippage) is a source of error when estimating joint kinematics of hindlimbs in rats from markers placed on the surface of the body overlying joints [56]. Therefore, mean distance between the hip marker and the knee marker or between the knee marker and the ankle marker were measured before and after lesion to the striatum. In the present study, locomotion without obstacle attached to the treadmill belt is referred to as stereotyped locomotion whereas locomotion with obstacles attached to the belt to as challenged locomotion.

### Numerical analysis

The step cycle was split into two parts, the stance and the swing phase. The stance phase was defined as the part of the cycle that begins as soon as the foot contacts the treadmill belt and terminates when the foot starts its forward movement (i.e. when the velocity of the MTP markers was higher than a threshold fixed at 5% of its maximal velocity). The swing phase was considered to begin at the onset of forward movement and to end when the foot strikes the treadmill belt. Using a MATLAB program (MathWorks, Natick, USA), we measured the following locomotor-related parameters:

- stance and swing phases duration, and stride duration (time in milliseconds between two successive foot contacts of the same limb),
- temporal symmetry ratio (TSR) of gait, a salient index of gait dysfunction in human stroke [57] was calculated for each of the locomotor cycles using the following equation: 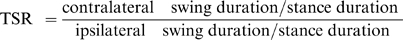

- stride length was computed as the Euclidian distance (mm) of the more distal markers (MTP for the hindlimbs, MTC for the forelimbs) between the beginning of the swing phase and the next contact with the treadmill belt. The reference frame was fixed to the hip marker,
- interlimb coordination. We calculated the homologous, homolateral and diagonal coupling from the time of the paw contact of a given limb with respect to the step cycle of the limb of the same girdle, of the same side, and of the diagonal limb, respectively,
- maximal (Max) and minimal (Min) values of joint angles during the stance and the swing phases,
- paw placement of the more distal marker of limbs at toe off in the frontal plan. For the hindlimb (see Fig.7C), this parameter corresponds to the position of the MCP marker (marker i) on the Z-axis (mediolateral) with respect to the Y-axis (vertical) that passes through the hip marker (marker f). For the forelimbs, it corresponds to the position of the MTP marker on the Z-axis with respect to the Y-axis that passes through the shoulder,
- horizontal head-on-trunk position. This parameter was assessed from the measurement of the roll angle, i.e. the angle between the straight line passing through the dorsal markers 1 and 2 and that passing through the dorsal markers 3 and 4 (see Fig.7D). A positive angle indicates a deviation of the head towards the right side,
- lateral tilt of the body. This parameter was assessed from the measurement of the lateral tilted angle, i.e. the angle between the horizontal plane of the laboratory and the plane passing through the two hip markers and the two shoulder markers (see Fig.7E). A positive angular value indicates a tilt toward the right side.

We also measured the following obstacle avoidance-related parameters:

- pre-obstacle distance: the distance between the obstacle and the tip of the paw just before the crossing swing,
- post-obstacle distance: the distance between the obstacle and the tip of the paw just at crossing swing ending,
- time to avoid obstacle, i.e. the duration of successful crossing swings (from the toe-off before obstacle to the paw contact after obstacle),
- the maximal height of the more distal marker during the crossing swing.

The parameters of stereotyped locomotion were calculated for 15 step cycles with at least four regular and consecutive step cycles during each trial in order to eliminate deviant curves [58]. The parameters used for assessing obstacle avoidance were calculated for 25 obstacle crossings with at least four consecutive crossings.

### Histological study

The lesion volume and the amount of histologically intact residual brain tissue were measured at the end of the experiment. After anaesthesia (chloral hydrate, 400 mg/kg, i.p.), rats were subjected to a transcardial perfusion with saline followed by a perfusion with paraformaldehyde (4% in phosphate buffer). Then, the brains were removed, postfixed for 30 min in paraformaldehyde, submerged for 36 h in 20% sucrose at 4°C, and frozen in isopentane (−40°C). Coronal sections (20 µm, 200 µm apart, and starting +2.2 mm to bregma and extending back to −3.6 mm to bregma) were collected on SuperFrost slides and stained with Cresyl violet (0.4%). Histological measurements were performed on sections using an image analyzing system (Scion Image, NIH, Bethesda, MD, USA). The areas of the lesion, the cavitations within parenchyma, the ventricles and the entire hemispheres were measured by contour tracing these regions on the computer screen. Corresponding volumes were calculated as the product of the sum of the areas and the distance between sections. Tissue loss induced by malonate poisoning corresponded to the difference in the amount of histologically intact residual tissue between the lesioned and the unlesioned hemispheres.

### Statistical analysis

Data are expressed as mean±SD. Statistics were performed using the 9.0 version of SYSTAT (Systat Software, Inc, Chicago, USA). Friedman's non parametric test was used to detect a global difference between kinematic recordings. If the P value was below 0.5, we compared data collected at days 7 and 60 post-lesion with those collected before lesion using Wilcoxon's test two times with Bonferroni's procedure. Such a small set of planned comparisons should increase only slightly the type I error risk as compared to more numerous planned comparisons. If these comparisons were both significant (P<0.025), it was concluded that lesions produced persistent impairment in kinematics. If only the comparison at day 7 post-lesion was significant (P<0.025), the impairment was suggested to regress over time. If only the comparison at day 60 post-lesion was significant (P<0.025), a delayed kinematic impairment was suggested.
